# Evaluation of the inflammatory response in a two-hit acute lung injury model using [^18^F]FDG microPET

**DOI:** 10.3892/etm.2013.1260

**Published:** 2013-08-13

**Authors:** GUANG-JU ZHOU, SHOU-YIN JIANG, MAO ZHANG, JIAN-XIN GAN, GUAN-YU JIANG

**Affiliations:** Department of Emergency Medicine, The Second Affiliated Hospital, Zhejiang University School of Medicine, Hangzhou, Zhejiang 310009, P.R. China

**Keywords:** acute lung injury, two-hit, lipopolysaccharide, [^18^F]fluorodeoxyglucose, mean arterial pressure

## Abstract

The aim of this study was to investigate whether a two-hit acute lung injury (ALI) model is better than a one-hit model in simulating ALI, and to evaluate the inflammatory response in the lungs in these two models using micro-positron emission tomography (microPET) with [^18^F]fluorodeoxyglucose (FDG). Sprague Dawley rats were divided into four groups; rats in the lipopolysaccharide (LPS; n=10) and LPS-HCl (n=10) groups were challenged by the intraperitoneal administration of 5 mg/kg LPS, while rats in the normal saline (NS; n=3) and HCl (n=10) groups received the same volume of normal saline solution. Sixteen hours following the administration, the rats in the HCl and LPS-HCl groups received an acid instillation (IT) of 0.5 ml/kg HCl (pH=1.2), while the rats in the remaining two groups received the same volume of normal saline solution. The mean arterial blood pressure (MAP) and blood gas concentrations were measured in all four groups. MicroPET was performed 4 h following HCl IT and the lungs were excised for histopathological examination. The rats in the LPS-HCl group exhibited a higher arterial PaO_2_ and a lower arterial PaCO_2_ compared with the rats in the remaining groups. The MAP decreased markedly in the LPS-HCl group, but remained stable in the LPS, HCl and NS groups. MicroPET results identified that the region of interest ratio in the LPS-HCl group (9.00±1.41) was significantly higher compared with those in the LPS (4.01±0.60) and HCl (3.33±0.55) groups (P<0.01). In addition, histological examination showed that the mean lung injury score in the LPS-HCl group (12.70±0.95) was significantly higher compared with those in the HCl (8.40±1.26) and LPS (7.00±0.82) groups (P<0.01). The present study demonstrates that LPS pretreatment significantly magnifies and prolongs the inflammatory response to subsequent acid IT in the lungs. Moreover, it is simpler to induce ALI using the two-hit model than with the one-hit model, and [^18^F]FDG microPET is a useful tool for evaluating the inflammatory reaction during ALI.

## Introduction

Acute lung injury (ALI) and its more severe form, acute respiratory distress syndrome (ARDS), are syndromes of acute respiratory failure that result from a variety of direct or indirect injuries to the lungs. When ALI or ARDS occur, there is a large influx of activated neutrophils into the lungs, proinflammatory mediators are produced and lung epithelial and endothelial surfaces are severely damaged (1). Although significant advances have been made in the treatment of these syndromes, mortality associated with ALI/ARDS remains high, at 30–70% (2). The complex pathogenesis of ALI makes animal models a necessity in the study of this disorder, and these models are numerous according to their insults of initiation, maintenance and host organisms utilized. Moreover, interpreting and applying the mechanistic data from animal models in the context of patients is challenging (3), and there is uncertainty as to which model best reflects the true situation in humans. However, numerous workshop participants have suggested that the two-hit model may be more appropriate for reflecting the common comorbidities and risk factors that are present in patients with ALI (4,5). The two-hit phenomenon indicates that an initial insult primes inflammatory cells so that a second insult results in an exaggerated response. However, other researchers consider that the one-hit model is more effective than the two-hit model, due to the reproducibility, rapid onset of clinical symptoms and lack of expense. Similarly, a previous study suggested that the introduction of a second hit has no impact on inflammation or increased lung injury (6). Therefore, based on previous findings, the present study aimed to investigate and compare the two models using [^18^F]fluorodeoxyglucose (FDG) micro-positron emission tomography (microPET) to evaluate the inflammatory response in the lungs in these models.

## Materials and methods

### 

#### Animals

Male Sprague Dawley rats (Grade II; weight, 180–210 g) were purchased from the Animal Center of Zhejiang University School of Medicine (Hangzhou, China). All animals were housed in air-filtered, temperature-controlled units with access to food and water *ad libitum*. All experimental protocols were approved by the animal care committee of Zhejiang University School of Medicine and the Principles of Laboratory Animal Care (NIH publication no. 86–23, revised 1985) were followed.

#### Experimental protocol

The rats were randomly divided into four groups; the rats in the lipopolysaccharide (LPS; n=10) and LPS-HCl (n=10) groups were challenged by the intraperitoneal (IP) administration of 5 mg/kg LPS (*Escherichia coli*, serotype 0111, B4; Sigma-Aldrich, St. Louis, MO, USA), while the rats in the normal saline (NS) control (n=3) and HCl (n=10) groups received an IP injection of 1 ml/kg normal saline solution. After 16 h, all animals were anesthetized with an IP injection of 40 mg/kg sodium pentobarbital and placed in a 60° inclined position. The femoral artery was cannulated and connected to a pressure transducer to record the arterial pressure and heart rate on a polygraph recorder (Shenzhen Mindray Bio-Medical Electronics Co. Ltd., Shenzhen, China). The trachea was surgically exposed, and the rats in the HCl and LPS-HCl groups received a direct intratracheal injection of 0.5 ml/kg HCl (pH=1.2), while rats in the NS control and LPS groups received the same volume of normal saline solution. Blood gas samples (0.3 ml) were obtained 30, 90 and 240 min following acid instillation (IT) and the lost blood was replaced by the same volume of saline solution. Samples were analyzed using a blood gas analyzer (Omni C; Roche Diagnostics, Indianapolis, IN, USA).

#### microPET examination

All rats underwent microPET examination 210 min following HCl IT. PET was performed using a microPET R4 rodent model scanner (CTI Concorde Microsystems Inc., Knoxville, TN, USA) that was equipped with microPET Manager for data acquisition in a list mode and ASI Pro VM™ software for preparing sinograms and image reconstruction. The scanner contained a computer-controlled bed and 10.8-cm transaxial and 7.8-cm axial fields of view (FOV) with an image resolution of <1.8 mm. FDG was prepared with a specific activity of 500 Ci/mmol in the Department of Nuclear Medicine, Zhejiang University School of Medicine (Hangzhou, China). Rats woke up 210 min after the instillation, so rats were reanesthetized in order to undergo microPET examination. Prior to examination, the rats were reanesthetized and injected with 10.56 MBq (0.3 mCi) FDG through the cannulation. After 30 min, the rats were placed under the central FOV of the microPET R4 scanner and underwent a 10 min static examination. The images were reconstructed by a maximum a posteriori probability algorithm. The ratio of the regions of interest (ROI) in the right lung to the muscle were calculated for each scan using ASI Pro VM. These ROIs were drawn and placed by one of the authors who had extensive experience in manual ROI definition and was blinded to the results. Corrections for dead time (time after each event during which the system is not able to record another event), random scattering and attenuation were performed for all scans.

#### Histology

At the end of the experiment, all rats were killed with an overdose of pentobarbital sodium. The left lung was placed in 4% formalin, embedded in paraffin and stained with hematoxylin and eosin. According to an arbitrary four-grade scale (7), all sections were examined and graded by a pathologist who was unaware of the experimental conditions of each animal. Briefly, the sections were assessed with regard to the airway epithelial necrosis, intra-alveolar edema, hyaline membranes, hemorrhage and recruitment of inflammatory cells to the air spaces. Each characteristic was scored between 0 and 3 (0, absent; 1, mild; 2, moderate; 3, prominent).

#### Statistical analysis

Data are expressed as the mean ± standard deviation and were analyzed using SPSS statistical software, version 17.0 (SPSS, Inc., Chicago, IL, USA). One-way analysis of variance with repeated measurement analysis was used to compare samples obtained at several time points from the same animals. An independent samples t-test was used to determine which groups were significantly different. P<0.05 was considered to indicate a statistically significant difference.

## Results

### Blood gas analysis

#### Arterial PaO_2_

**Figure 1.**
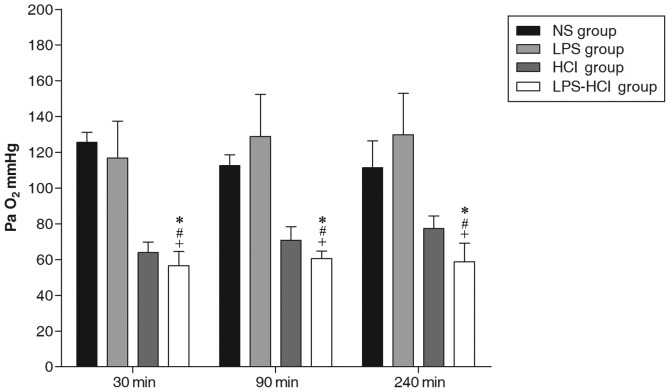
Changes in the PaO_2_ in the NS (n=3), LPS, HCl and LPS-HCl groups (n=10 for each). Data are presented as the mean ± SD. ^#^P<0.05 vs. the NS group;^*^P<0.05 vs. the LPS group; ^+^P<0.05 vs. the HCl group. NS, normal saline; LPS, lipopolysaccharide.

The arterial PaO_2_ in the NS (116.54±10.97 mmHg) and LPS (125.20±22.49 mmHg) groups remained normal throughout this experiment. As expected, the PaO_2_ showed an initial decline in the HCl and LPS-HCl groups following acid IT. The average PaO_2_ was 58.67±7.77 mmHg in the LPS-HCl group and 70.68±8.67 mmHg in the HCl group (P<0.01). At the end point of the experiment, the mean PaO_2_ in the HCl group (77.29±7.15 mmHg) was higher compared with that of the LPS-HCl group (58.81±10.27 mmHg) and the difference between the two groups was statistically significant (P<0.01; Fig. 1).

#### Arterial PaCO_2_

**Figure 2.**
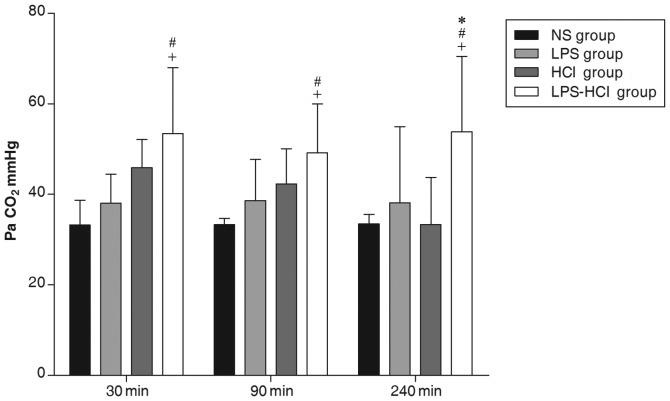
Changes in PaCO_2_ in the NS (n=3), LPS, HCl and LPS-HCl groups (n=10 for each). Data are presented as the mean ± SD. ^#^P<0.05 vs. the NS group;^*^P<0.05 vs. the LPS group; ^+^P<0.05 vs. the HCl group. NS, normal saline; LPS, lipopolysaccharide.

The arterial PaCO_2_ in the LPS-HCl group (52.14±13.86 mmHg) was significantly higher compared with those in the HCl (41.17±9.18 mmHg), LPS (38.25±11.24 mmHg) and NS control (33.32±3.02 mmHg) groups (P<0.05; Fig. 2).

#### Mean arterial pressure (MAP)

**Figure 3.**
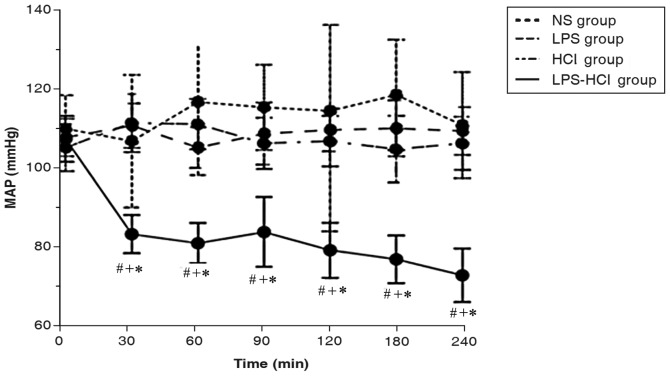
MAP measurements in the NS (n=3), LPS, HCl and LPS-HCl groups (n=10 for each). Data are presented as the mean ± SD. ^#^P<0.05 vs. the NS group; ^*^P<0.05 vs. the LPS group; ^+^P<0.05 vs. the HCl group. MAP, mean arterial pressure; NS, normal saline; LPS, lipopolysaccharide.

Basal measurements of the MAP among the four groups were not different. The MAP in the LPS-HCl group decreased markedly while the MAP in the LPS, HCl and NS control groups remained stable. The MAP of the LPS-HCl group was significantly different compared with those of the other three groups at the subsequent time points (P<0.001; Fig. 3).

#### microPET

**Figure 4.**
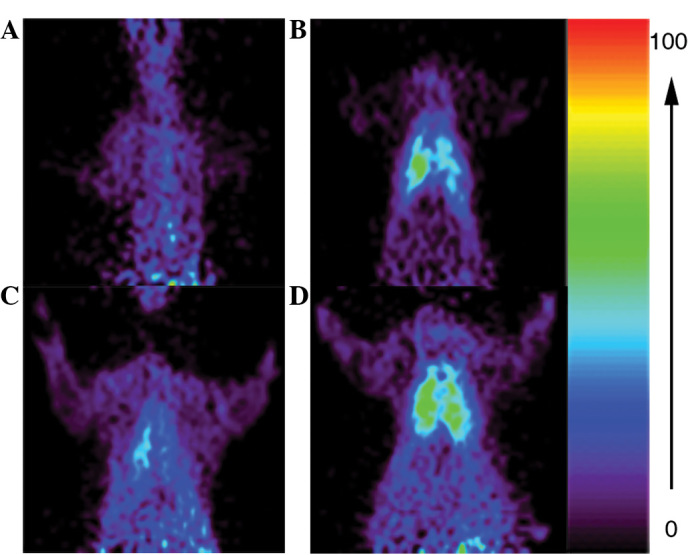
microPET scans of rats in the (A) NS; (B) LPS; (C) HCl and (D) LPS-HCl groups. The rats in LPS-HCl group displayed higher (^18^F)FDG uptake than the rats in the other groups. PET, positron emission tomography; NS, normal saline; LPS, lipopolysaccharide; FDG, fluorodeoxyglucose.

The ratios of mean [^18^F]FDG uptake in the lungs to that in the muscle tissue were compared among the different groups of rats and the ratio in the LPS-HCl group (9.00±1.41) was observed to be significantly higher than the uptakes in the LPS (4.01±0.60) and HCl (3.33±0.55) groups (P<0.01; Fig. 4).

#### Histology

**Figure 5.**
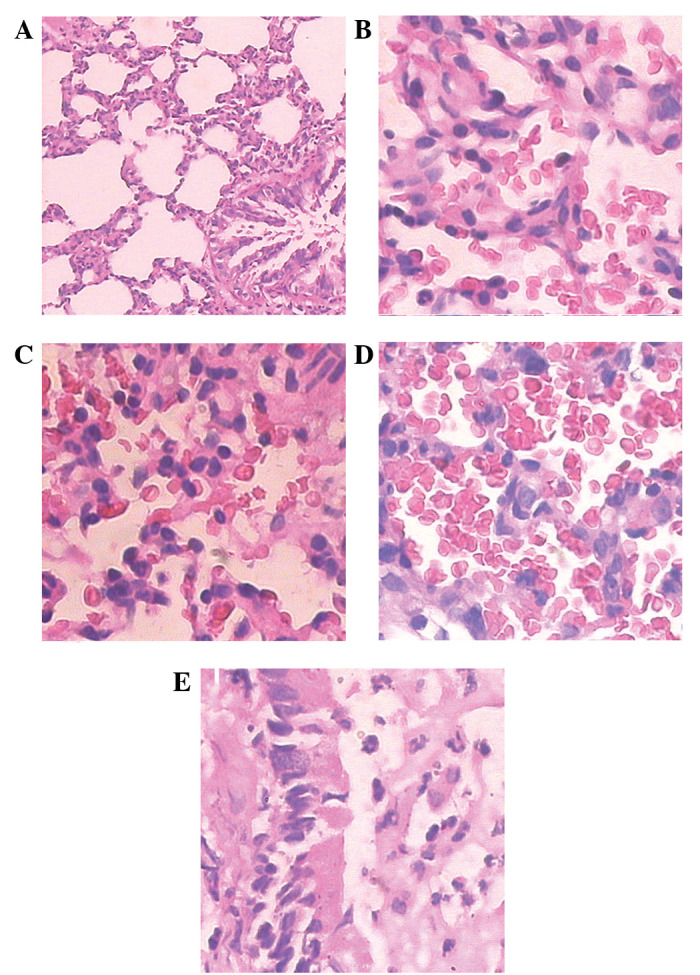
Histological examination of the lungs by hematoxylin and eosin staining. (A) NS (magnification, ×100), (B) LPS, (C) HCl and (D and E) LPS-HCl groups (magnification, ×400 for each). The LPS-HCl group displayed more bleeding and neutrophil infiltration than the other groups. NS, normal saline; LPS, lipopolysaccharide.

Histological examination showed that lung injuries of varying degrees occurred in all animals that had received LPS injection, HCl IT or both. Neutrophil infiltration and hemorrhage were present in all animals of the LPS, HCl and LPS-HCl groups, however, they were more prominent in the LPS-HCl group. Hyaline membranes were common in the LPS and LPS-HCl groups but were rare in the HCl group. Alveolar edema and airway epithelial necrosis were common and prominent in the HCl and LPS-HCl groups, while there were inconsistent findings in the LPS group. Briefly, the mean lung injury score in the LPS-HCl group (12.7±0.95) was significantly higher compared with the scores in the HCl (8.40±1.26) and LPS (7.00±0.82) groups (P<0.001; Fig. 5).

## Discussion

Although PET with [^18^F]FDG has become an established diagnostic tool in oncology in clinical practice, FDG PET is also emerging as a promising imaging technique for infectious and inflammatory diseases (8–10). [^18^F]FDG-labeled leukocyte PET/computed tomography (CT) has a high sensitivity and specificity for the diagnosis of infection. It may also accurately localize the foci of infection and the source of a fever of undetermined origin, thereby guiding additional testing (11). In ALI, the predominant inflammatory cells are neutrophils, and the adhesion and activation of neutrophils are important for the development of ALI (12,13). The activated neutrophils also take up [^18^F]FDG at an accelerated rate following [^18^F]FDG injection, thus generating a PET imaging signal. [^18^F]FDG PET/CT is a useful tool for evaluating pulmonary lesions, as FDG uptake may be quantified. The present study used FDG quantification to assess activated neutrophils and obtain satisfactory results.

Animal models mimicking human ALI have been useful for providing valuable information regarding the mechanisms underlying the pathogenesis of this injury. An increasing number of studies have suggested that LPS administration induces an inflammatory response in the lung in animal models (14–16) these effects are induced by the direct injury of endothelial cells and by the activation of inflammatory cells, and has been considered to be an appropriate model for ALI experiments (17). However, the LPS-induced lung injury model in animals has its limitations in reflecting the true situation in human patients with ALI or ARDS. Clinically, the development of ALI and ARDS is complex and it is rare for these conditions to be caused by only a single instigating factor (18). The occurrence of ALI increases with multiple risk factors and direct pulmonary disorders (such as pneumonia, aspiration or pulmonary contusion) and indirect pulmonary risk factors (such as sepsis or multiple trauma) are well-known risk constellations (19).

A synergistic response has previously been shown in the two-hit model. Studies have demonstrated that in animal models, neutrophil recruitment to the lungs is enhanced when hemorrhagic shock is followed by LPS treatment and when sepsis is followed by direct lung injury with immune complexes or LPS (20,21,22). In the present study, [^18^F]FDG microPET showed that rats in the LPS-HCl group had a significant influx of activated neutrophils into the lungs, whereas rats in the LPS and HCl groups exhibited lower level of influx. In addition, pretreatment with LPS significantly increased and prolonged the reduction in arterial PaO_2_. These results indicate that the IP injection of LPS greatly increased the inflammatory response to acid IT and caused the rats to become more susceptible to ALI.

The histological examination results also demonstrated that LPS pretreatment significantly magnified the inflammatory response to acid aspiration. Neutrophil infiltration, hemorrhage, hyaline membranes, alveolar edema and airway epithelial necrosis were observed to be common and prominent in the two-hit model; however, there were inconsistent findings in the LPS and HCl groups. The results showed that the uptake of [^18^F]FDG examined by microPET positively correlated with the histopathological lung injury score (R=0.831, P<0.01). Thus, we conclude that by using [^18^F]FDG PET, it is possible to accurately assess the degree of lung injury.

The findings of the present study may be explained by the two-hit model. In this model, an initial insult primes the host to generate an amplified response to a second insult. The initial hit (LPS IP injection) predisposed the rats to produce an augmented inflammatory response to the second hit (acid IT). The same dose of LPS or HCl alone only resulted in a localized inflammatory response. However, the combination of LPS and HCl generated a generalized inflammatory response that was detected in both lungs.

As infection is often associated with other risk factors in the clinic, this two-hit ALI model reflects a potential clinical situation more effectively than a one-hit model. Therefore, by closely paralleling the clinical development of pulmonary injury, the two-hit model may be invaluable for the study of human ALI.
